# Chronopharmacodynamics and Chronopharmacokinetics of Pethidine in Mice

**DOI:** 10.1371/journal.pone.0102054

**Published:** 2014-07-15

**Authors:** Chengliang Zhang, Zaoqin Yu, Xiping Li, Yanjiao Xu, Dong Liu

**Affiliations:** 1 Department of Pharmacy, Tongji Hospital, Tongji Medical College, Huazhong University of Science and Technology, Wuhan, China; 2 Department of Clinical Pharmacy, College of Pharmacology, China Pharmaceutical University, Nanjing, China; Karlsruhe Institute of Technology, Germany

## Abstract

**Background:**

Many studies have demonstrated that the pharmacokinetics and pharmacodynamics of analgesic drugs vary according to the circadian time of drug administration. This study aims at determining whether the analgesic effect and pharmacokinetics of pethidine in male BALB/c mice are influenced by administration time.

**Methods:**

A hot-plate test was used to evaluate the analgesic effect after pethidine (20 mg/kg) or saline injection at different dosing times. Mouse blood samples were collected at different intervals after dosing at 9:00 am and 9:00 pm, and were determined via liquid chromatography–tandem mass spectrometry (LC–MS/MS).

**Results:**

A significant 24-h rhythm was observed in the latency to thermal response at 30 min after dosing, with the peak during the dark phase and the nadir during the light phase. Tolerance to analgesic effect was produced after chronic pethidine injection at 9:00 am or 9:00 pm, and the recovery from tolerance was faster during the dark phase. The peak concentration (C_max_) and area under the concentration–time curve (AUC) of pethidine and its metabolite norpethidine were significantly higher during the dark phase than during the light phase, but the total serum clearance (CL/F) exhibited the opposite trend. The rhythm of drug plasma concentration was positively correlated with the analgesic effect.

**Conclusion:**

These results suggest that the pharmacodynamics and pharmacokinetics of pethidine in mice vary significantly according to the dosing time, which implies that the time of administration should be considered in the rational clinical use of pethidine to maximise analgesia and minimise the adverse effects.

## Introduction

Numerous physiologic and biochemical processes in animals display biological rhythms, including heart rate, blood pressure, body temperature and hormone secretion [1]–[3]. Circadian rhythm is controlled by an inherent molecular clock network composed of the paired suprachiasmatic nuclei (SCN), which are situated in the hypothalamus and the pineal gland [4], [5]. Large numbers of drugs, such as antibiotics [6], antitumor drugs [7], cardiovascular drugs [8], immunosuppressive agents [9], antidepressants [10] and analgesics [11], [12], also vary significantly in potency and disposition kinetics depending on the time of drug administration.

Pain is a very complex and subjective phenomenon, and treatment for chronic and acute pain is often inadequate in many clinical situations. Circadian variations with respect to the occurrence and intensity of pain have been observed in animals and humans [13]. Chronic pain and acute pain have different rhythm patterns, and significant inter-individual differences have been described in patients. These findings suggest that the time of analgesic administration should be consistent with the circadian pattern of pain intensity to maximize relief and control pain (i.e., chronotherapy).

Pethidine is a synthetic narcotic analgesic predominating μ-opioid receptor, which is widely used to relieve moderate-to-severe pain and control post-anaesthetic shivering [14]. However, similar to other opioids, pethidine may cause physical dependence or addiction after long-term use. Pethidine is metabolised in the liver via two distinct pathways. The most clinically significant pathway is N-demethylation by the hepatic cytochrome P-450 system into norpethidine, a non-opioid active metabolite [15]. Norpethidine has half the analgesic potency of pethidine; however, norpethidine has two to three times the potency of pethidine as a central nervous system (CNS) excitatory agent; it can cause delirium, tremulousness, hallucinations, hyperreflexia and convulsions [16]. Over the past few decades, considerable studies have reported the pharmacodynamics and pharmacokinetics of pethidine in humans, as well as in animals [17]–[20]. However, few studies have investigated whether the analgesic effect and pharmacokinetics of pethidine are related to the circadian time of pethidine administration.

The objective of the present study was to observe the influence of dosing time on the pharmacodynamics and pharmacokinetics of pethidine in BALB/c mice. This study reveals a significant 24-h rhythm in the latency to thermal stimulus after pethidine injection, and pharmacokinetics of pethidine and norpethidine clearly varied according to the time of administration. The dosing time-dependent variation of plasma pethidine concentration was positively correlated with that of pain relief.

## Materials and Methods

### Animals

Male BALB/c mice weighing 18 g to 22 g were purchased from the Experiment Animal Center of Tongji Medical College, Huazhong University of Science and Technology (Wuhan, China). Mice were housed under standardised light-controlled conditions (lights on from 7:00 am to 7:00 pm) at room temperature (24±1°C) and 50±10% humidity, with free access to food and water. All mice were adapted to their light/dark cycle for at least one week before the experiment. Moreover, the dim red light was used to treatment of mice during the dark phase. The experiments were performed with formal approval from the Animal Ethics Committee of Tongji Medical College, Huazhong University of Science and Technology. The animals were handled in accordance with the Guide for the Care and Use of Laboratory Animals of the National Institutes of Health.

### The 24-h rhythm of analgesic effect

To observe the 24 h rhythm of the analgesic effect of pethidine, the mice in each group (n = 10) were given a single i.p. dose of 20 mg/kg pethidine (Qinghai Pharmaceutical factory Co., Ltd., Xining, China) or saline at the following time points: 9:00 am, 1:00 pm, 5:00 pm, 9:00 pm, 1:00 am and 5:00 am. The analgesic effect was evaluated after pethidine and saline injection using a hot-plate analgesia meter YLS-6B (Device Station in Shandong Academy of Medical Sciences, China). The plate surface temperature was set and maintained at 55±0.2°C, and the analgesic effect of pethidine was determined at 30 min after drug injection. Time (in seconds) to either hind paw licking or jumping was recorded as the pain response latency. The cut-off time was set at 60 s to prevent tissue damage [21].

### Tolerance and its recovery after chronic pethidine injection

**Figure 1:**
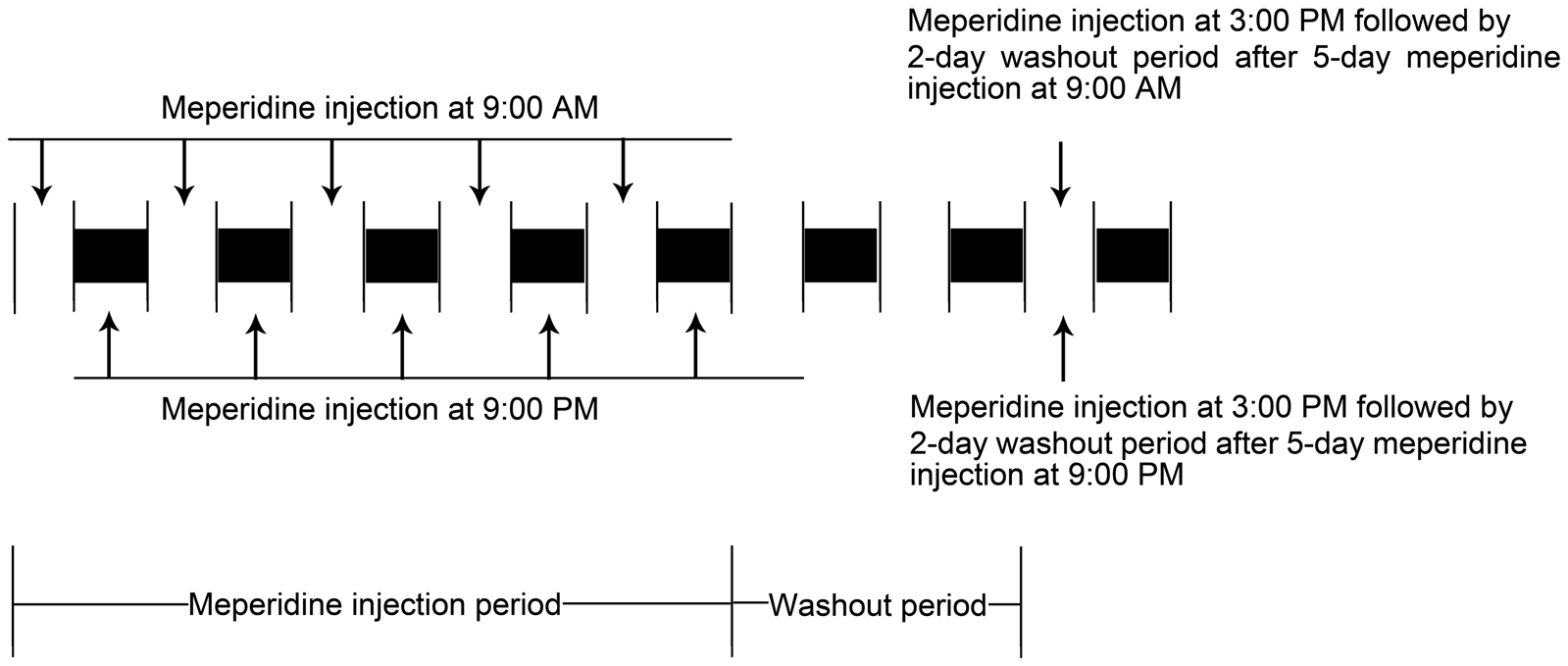
Time schedule of meperidine injection. Full legend: Time schedule of meperidine injection at 9:00 am or 9:00 pm for 5 days and then a 2-day washout period.

To observe the influence of dosing time on tolerance and recovery from tolerance after chronic pethidine injection (Figure 1, time schedule for the pethidine injection at 9:00 am or 9:00 pm for 5 days followed by a 2-day washout period), the mice in each group (n = 10) were given a single i.p. dose of pethidine or saline at 9:00 am or 9:00 pm for 5 days. The analgesic effect of pethidine was determined daily 30 min after pethidine or saline injection. After a 2-day washout period, recovery from the analgesia was measured 30 min after a single pethidine injection at 3:00 pm.

### Chronopharmacokinetics of pethidine and its metabolite

To investigate the pharmacokinetics of pethidine, all mice were randomly assigned to two groups. The mice in each group (n = 84) were given a single i.p. injection of pethidine (20 mg/kg) either at 9:00 am or 9:00 pm. Blood samples (0.5 mL) were respectively collected from the heart of seven mice at different time intervals after pethidine injection (e.g., 0.08, 0.17, 0.25, 0.33, 0.50, 0.75, 1, 1.5, 2, 2.5, 3 and 4 h), and then the mice were sacrificed by cervical dislocation under anesthesia. Plasma specimens were centrifuged at 3,000 rpm for 15 min, and the supernatant was subsequently removed and immediately frozen at −80°C until analysis.

### Determination of pethidine and its metabolite

Pethidine and norpethidine concentrations were determined according to a previously described liquid chromatography–electrospray ion (ESI) source–tandem mass spectrometry (LC–ESI–MS/MS) method applied in our laboratory [22]. The pharmacokinetic parameters were estimated using a compartment model via the DAS software package. The peak plasma concentration (C_max_) and the time to reach peak concentration (T_max_) were directly observed from the plasma concentration–time profiles. The area under each drug plasma concentration–time curve (AUC) was calculated by applying the linear trapezoidal rule. Terminal elimination half-life (t_1/2_z) was calculated using the equation t_1/2_ = 0.693/kel, where kel is the terminal elimination rate constant determined via linear regression analysis of the slope of the terminal phase of the log–linear plasma concentration–time curve. The apparent total clearance (Cl/F) was calculated as dose/AUC, and the apparent volume of distribution (V/F) as Cl/kel.

### Statistical analysis

The pharmacodynamics data are presented as mean ± SD, whereas the pharmacokinetic data are presented as mean. Comparisons were performed by a one-way analysis of variance (ANOVA) and repeated ANOVA, and differences between groups were determined using Scheffe's test. A probability level of less than 0.05 was considered significant.

## Results

### The 24-h rhythm of the analgesic effect

**Figure 2:**
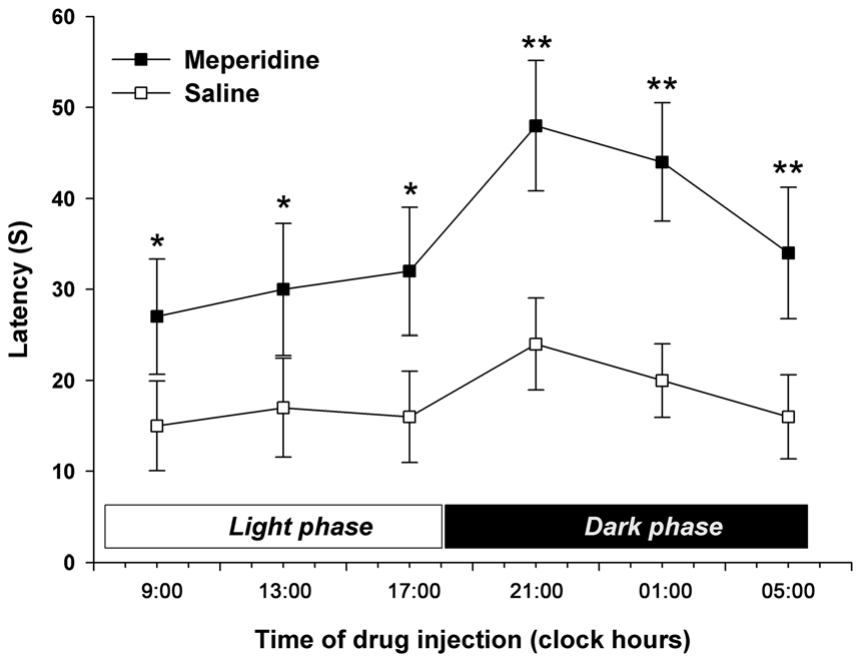
The 24-h rhythm of analgesic effect after meperidine injection. Full legend: The 24 h rhythm of analgesic effect after meperidine (20 mg/kg i.p.) (▪) or saline (□) injection. Each value represents the mean with SD. ^*^ *P*<0.05, ^**^ *P*<0.01 compared with the saline group using Scheffe's test.

The latency to thermal response after saline injection was longer during the dark phase than during the light phase (*P*<0.01). The analgesic effect after pethidine injection was significantly stronger than that after saline injection during the dark and light phases (light phase, *P*<0.05; dark phase, *P*<0.01; Figure 2). A significant 24-h rhythm in the latency to thermal response was also observed after pethidine injection, with the peak during the dark phase and the nadir during the light phase (*P*<0.01).

### Tolerance and recovery after chronic pethidine injection

**Figure 3:**
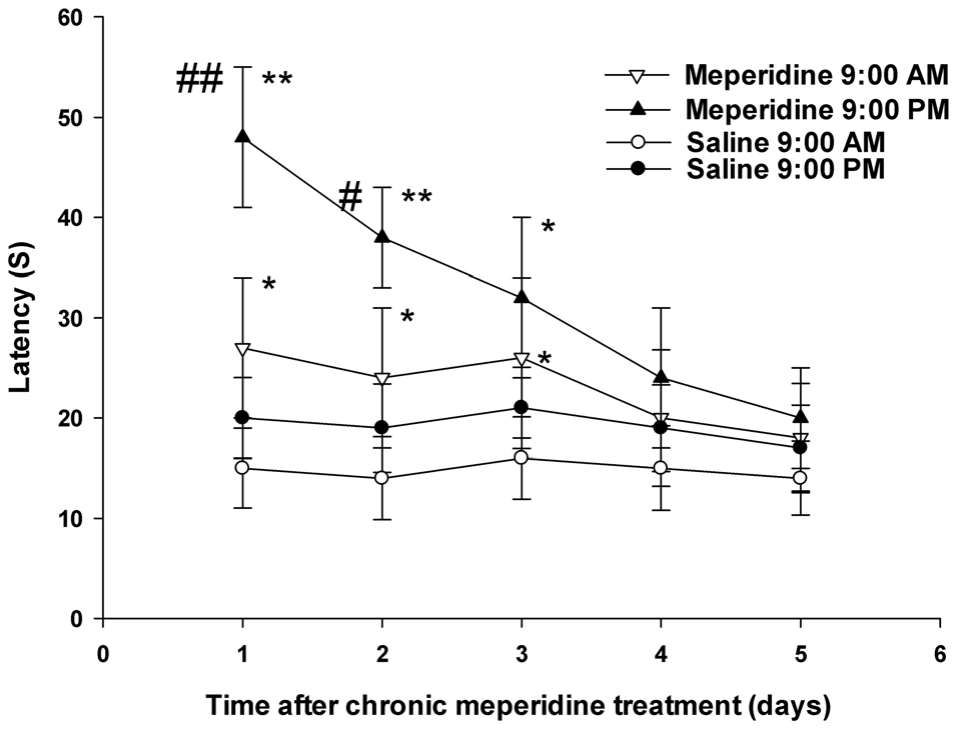
Influence of dosing time on tolerance after 5-day meperidine injection at day or night. Full legend: Influence of dosing time on tolerance to analgesia at 30 min after 5-day meperidine or saline injection at 9:00 am or 9:00 pm. Each value represents the mean with SD. ^*^ *P*<0.05, ^**^ *P*<0.01 compared with the corresponding saline group, and ^#^ *P*<0.05, ^##^ *P*<0.01 comparison between two dosing time using Scheffe's test.

Tolerance to analgesia after daily pethidine or saline injection at 9:00 am or 9:00 pm for 5 days is illustrated in Figure 3. For the saline group, no statistical difference in pain threshold was observed after the 5-day injection at 9:00 am or 9:00 pm (*P*>0.05). In the first three days, the analgesic effect either at 9:00 am or 9:00 pm after pethidine injection was significantly stronger than that after saline injection (9:00 am, *P*<0.05; 9:00 pm, days 1 and 2, *P*<0.01, day 3, *P*<0.05). On days 1 and 2, the pain threshold was higher after pethidine injection at 9:00 pm than 9:00 am (day1, *P*<0.01; day 2, *P*<0.05). Tolerance to the analgesic effect after pethidine injection at 9:00 am or 9:00 pm was developed on days 4 and 5. Ultimately, no apparent difference was observed compared with that after the corresponding saline injection (*P*>0.05); however, the analgesic effect of pethidine was relatively stronger during the dark phase in the first 3 days.

**Figure 4:**
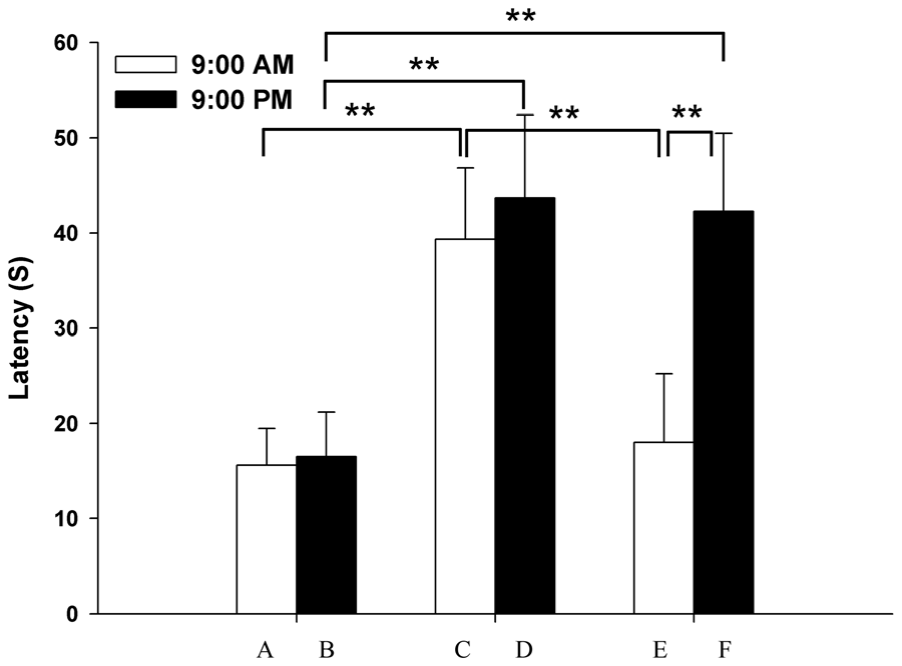
Influence of dosing time on recovery from tolerance after 2-day washout period. Full legend: Influence of dosing time on recovery from tolerance after a single meperidine (20 mg/kg i.p.) or saline injection at 3:00 pm, followed by a 2-day washout period after 5-day meperidine or saline injection at 9:00 am or 9:00 pm (□, 9:00 am; ▪, 9:00 pm). Each value represents the mean with SD. ^*^ *P*<0.05, ^**^ *P*<0.01 comparison between groups using Scheffe's test. A, a single saline injection after 5-day saline injection at 9:00 am. B, a single saline injection after 5-day saline injection at 9:00 pm. C, a single meperidine injection after 5-day saline injection at 9:00 am. D, a single meperidine injection after 5-day saline injection at 9:00 pm. E, a single meperidine injection after 5-day meperidine injection at 9:00 am. F, a single meperidine injection after 5-day meperidine injection at 9:00 pm.

Recovery from tolerance, followed by a 2-day washout period after a 5-day pethidine treatment, is shown in Figure 4. No significant difference was noted in the latency to thermal stimulus of either single saline or pethidine injection at 3:00 pm, which was followed by washout after saline injection at 9:00 am or 9:00 pm for five days (*P*>0.05). Undergoing a 2-day washout period indicated that the analgesic effect at 3:00 pm after pethidine injection for the 5-day administration at 9:00 pm was clearly stronger than that in the corresponding saline group (*P*<0.01); however, such result was not observed in the 5-day pethidine injection at 9:00 am (*P*>0.05). Pain latency after pethidine injection at 3:00 pm was significantly longer for the 5-day pethidine injection at 9:00 pm than at 9:00 am (*P*<0.01).

### Chronopharmacokinetics of pethidine and its metabolite

**Figure 5:**
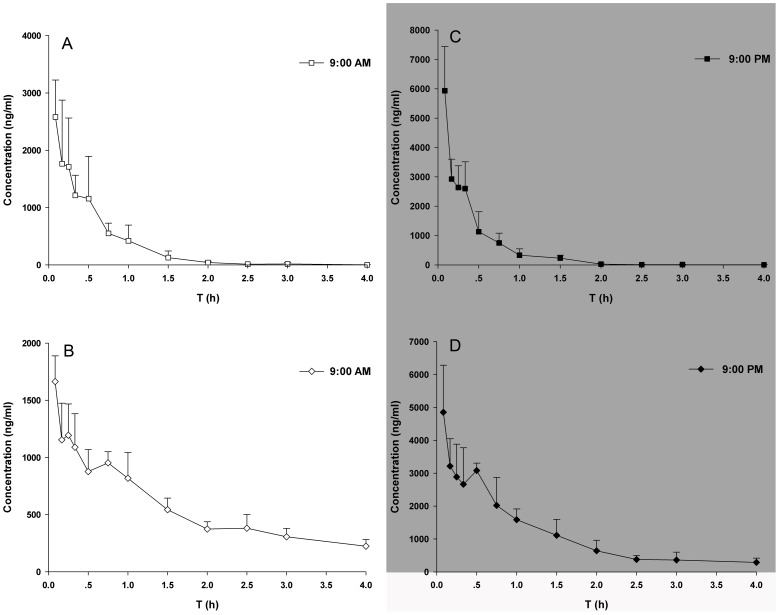
Plasma concentration-time profiles after meperidine injection (20 mg/kg, i.p.) at 9:00 am and 9:00 pm. (**A, C**) Plasma concentration-time profiles of meperidine at 9:00 am and 9:00 pm. (**B, D**) Plasma concentration-time profiles of normeperidine at 9:00 am and 9:00 pm. Each value represents the mean. (n = 7).

Figure 5 shows the plasma concentration-time profiles of pethidine (Figure 5: A and C) and norpethidine (Figure 5: B and D) after a single i.p. pethidine injection (20 mg/kg) at 9:00 am or 9:00 pm. The plasma concentrations of pethidine and norpethidine during the light and dark phases displayed significant dosing time dependence. The pharmacokinetic parameters for each treatment–time group are presented in Table 1. As shown, the C_max_ and AUC for pethidine and norpethidine were obviously greater at 9:00 pm than at 9:00 am (*P*<0.01), whereas the CL/F was significantly lower at 9:00 pm than at 9:00 am (*P*<0.01).

**Table 1:** Parameters of meperidine and normeperidine after meperidine (20 mg·kg^−1^, i.p.) injection at 9:00 am or 9:00 pm.

Parameters	Meperidine		Normeperidine	
	9:00AM	9:00PM	9:00AM	9:00PM
T_max_(h)	0.21	0.12	0.18	0.167
C_max_(ng·mL^−1^)	2736.31	5948.60**	1782.92	5087.02**
AUC(ng·h·mL^−1^)	1295.65	1976.74**	2619.19	5078.09**
CL/F(L·h^−1^)	15.92	10.35**	7.64	3.74**
T_1/2z_(h)	3.11	2.23	1.10	1.94
V/F(L·kg^−1^)	59.87	32.49	12.15	10.41

**P<0.01 comparison between two dosing times.

T_max_: time to reach peak concentration; C_max_: peak concentration; AUC: area under the plasma concentration-time curve from zero to time infinity; CL/F: apparent total clearance; T_1/2z_: Terminal elimination half-life; V/F: apparent volume of distribution; F: bioavailability (mean, n = 7).

## Discussion

In the present study, we observed a significant 24-h rhythm in the latency to thermal stimulus after pethidine injection, with a peak during the dark phase and a nadir during the light phase. Therefore, rhythm of analgesic effect induced by pethidine resembled the inherent pain rhythm in animals. This result suggests that analgesic rhythm of pethidine is similar to that induced by morphine which exerted strong analgesic effect during the dark phase, in accordance with rhythmic expreesion of morphine μ-opioid receptor [23].

Previous study has shown that pethidine given alone (8, 16, 24 mg/kg, s.c.) in mice by tail-flick test produced antinociception in a dose-dependent manner, and pethidine (24 mg/kg, s.c.) alone produced a maximum antinociception [24]. However, severe sedation was observed after s.c. injection of more than 30 mg/kg pethidine in mice [25]. Dosage of pethidine hydrochloride used for the evaluation of analgesic effect was ultimately chosen to 20 mg/kg in our experiment after several dose explorations.

Pethidine is similar to other opioids, such as morphine, which can potentiate tolerance after long-term use. This study showed that analgesic effect on days 4 and 5 after pethidine injection at 9:00 pm or at 9:00 am was apparently decreased and had no significant dose–time dependent difference compared with that after the corresponding saline injection. Therefore, tolerance to analgesic effect is gradually produced after chronic pethidine injection. The mechanism with respect to development of tolerance may be associated with the downregulation of μ-opioid receptors. Pethidine is a μ-opioid receptor agonist with a lower intrinsic activity than morphine [26]. Studies have confirmed that pethidine produces profound tolerance and the magnitude of tolerance varies inversely with intrinsic activity; however, is presumably related directly to receptor occupancy [27], [28]. Specifically, receptor occupancy functions in the development of tolerance, resulting in decrease of μ-opioid receptor expression, and a drug with low intrinsic activity produces higher tolerance than a drug with high intrinsic activity. Besides, pethidine is an atypical opioid which bears NMDA (N-methyl-D-aspartate) receptor antagonist, anticholinergic and local anesthetic properties along with the prominent μ- and κ-agonistic effects [15], [29], [30]. Some of those effects in its properties may influence the process of tolerance development.

In the current study, we found a dosing time–dependent difference in the recovery from tolerance after chronic pethidine injection; however, statistical difference was not demonstrated between 9:00 am and 9:00 pm during the initial determination of the 24-h rhythm pattern. The recovery time from tolerance to analgesia was set to 3:00 pm to avoid a dosing time dependent effect, which was supported by the previous finding [31]. They verified that morphine administered at 3:00 pm did not alter the peak locomotor activity or body temperature within 9 h to 12 h, unlike the effects of morphine 9 h to 12 h after administration at 9:00 am. The washout period was designed for 2 days between the two dosing times to ensure that pethidine was almost completely eliminated. Namely, the washout time is 78 hours for the morning treatment group and 66 hours for the evening treatment group. Nevertheless, a rapid recovery from tolerance was defined for pethidine injection at 9:00 pm than 9:00 am. Definite mechanism involved in the recovery of tolerance is unclear and requires our further study.

Pethidine is mainly metabolised in the liver through hydrolysis into meperidinic acid and through N-demethylation into norpethidine, which is subsequently hydrolysed into normeperidinic acid [32]. The active metabolite of pethidine norpethidine has weak analgesic activity but a potent CNS excitotoxic effect, with clinical signs ranging from agitation to seizures. Studies have demonstrated that the severity of CNS excitation with pethidine administration is correlated directly with norpethidine levels [33], [34]. Our study observed significant dosing-time dependence in the pharmacokinetics of pethidine and norpethidine in mice, with higher C_max_, AUC and lower CL/F during the dark phase than that during the light phase. Therefore, the rhythm pattern in the pharmacokinetics of norpethidine is identical to that of pethidine, which indicates the possible accumulation of norpethidine during the dark phase that causes toxicity and adverse reactions. These findings suggest that the possible neurotoxicity induced by norpethidine should be paid more attention when pethidine is administered during the dark phase.

The study describes a positive correlation between the rhythms of plasma pethidine and norpethidine concentrations and analgesic effect during the dark and light phase. Specifically, the greater analgesic effect of pethidine coincides with higher plasma concentration, which guides the best dosing regimen for the rational use of pethidine. In one human study, pethidine efficacy significantly differed between day and night treatment groups, with the elimination half-time (T_1/2_β) 46% shorter and the total serum clearance 70% greater during the night time. Drug concentration was positively correlated with pain relief in the day group, but no such correlation was observed in the night group [35]. Many studies have shown that human rest/activity cycles contrast with those of rodents, especially mice. For instance, analgesic effect induced by tramadol was strongest at the end of mouse activity period (i.e., early morning) [36], whereas healthy volunteers experienced stronger analgesic sensitivity when the drug was administered in the early evening [37]. Therefore, to some extend, the results in mice are supported by the previous human study, with higher peak concentration and stronger analgesic effect during the dark phase.

## Conclusions

Overall, our study demonstrates the dosing time–dependent differences in the pharmacodynamics and pharmacokinetics of pethidine in mice. The dosing time dependence in the pethidine concentration–time profile is correlated with pain relief. This finding implies that pethidine administration time should be considered during pain treatment to maximise analgesia, reduce side effects and improve patient compliance. Moreover, during the dark phase, the analgesic effect of pethidine was greater when the drug plasma level was higher; however, the toxicity induced by its metabolite norpethidine might be stronger. Therefore, further studies should be combined with clinical practice.
